# Translation and validation of Berlin questionnaire in primary health care in Greece

**DOI:** 10.1186/1471-2466-13-6

**Published:** 2013-01-24

**Authors:** Izolde Bouloukaki, Ioannis D Komninos, Charalampos Mermigkis, Katerina Micheli, Maria Komninou, Violeta Moniaki, Eleni Mauroudi, Nikolaos M Siafakas, Sophia E Schiza

**Affiliations:** 1 Department of Thoracic Medicine, Sleep Disorders Unit, University of Crete, Heraklion, Crete, Greece; 2 Vrachasi Practice, General Hospital/Health Centre of Neapolis, Heraklion, Crete, Greece; 3 Department of Social Medicine, University of Crete, Heraklion, Greece

**Keywords:** Berlin questionnaire, Obstructive sleep apnoea syndrome, Primary care patients, Screening tool, Greece, validation

## Abstract

**Background:**

The aim of our study was to validate a Greek translation of the Berlin Questionnaire (BQ) for obstructive sleep apnoea syndrome (OSAS) and to explore whether this screening questionnaire could be used to help identify primary care patients at greater risk of having OSAS.

**Methods:**

We recruited 189 patients visiting a primary health care setting on the island of Crete, Greece. They all completed the Greek Version of the BQ. Patients were then referred to a Sleep Disorders Unit for evaluation of suspected sleep-disordered breathing.

**Results:**

A PSG study was performed in 129 of the 189 subjects (68.3%). BQ identified 74.4% (n = 96) of the patients as high-risk for OSAS and the remaining 25.6% (n = 33) as low-risk. The sensitivity and specificity of BQ for OSAS diagnosis were 76% and 40%, respectively, for an apnoea–hypopnoea index (AHI) ≥5 per hour but <15 per hour, 84% and 61% for an AHI ≥15 per hour but ≤30 per hour, and 79% and 39% for an AHI >30 per hour.

**Conclusions:**

In conclusion, the Greek Version of the BQ is a useful instrument for identifying patients at risk for OSAS in primary health care in Greece. The findings of our study confirm that such screening tools should be used by primary care clinicians for OSAS prediction.

## Background

Obstructive sleep apnoea syndrome (OSAS) is a common disease that is characterised by repetitive episodes of partial or complete upper-airway obstruction during sleep, leading to adverse consequences for both health and quality of life [1-5]. It is currently estimated that at least 1 in 5 American adults has some degree of sleep apnoea, while 1 in 15 has moderate or severe disease [6]. Most epidemiological studies of the prevalence of obstructive sleep apnoea have been performed in North America, Europe, or Australia. However, as the awareness that OSAS can lead to serious medical sequelae is increasing, several studies have been undertaken to estimate the disease burden in other countries, such as China, India, and Korea. Unfortunately, studies investigating the prevalence of OSAS in Greece are lacking. Furthermore, the majority of patients with OSAS remain undiagnosed. In fact, in the Wisconsin Sleep Cohort Study, it was estimated that 93% of women and 82% of men with moderate to severe OSAS are undiagnosed [7]. Therefore, as our understanding of the prevalence and pathophysiology of OSAS expands, clinicians should evaluate patients with OSAS with a view to preventing these potential consequences.

The gold standard for the diagnosis of OSAS is attended overnight polysomnography (PSG) [8]. A reliable and easy-to-use screening tool is required for efficient prediction of OSAS, helping to prioritize patients who require sleep study according to the probability of suffering from OSAS. Such a screening tool would give treatment to those who are with more severe OSAS first. Meanwhile, it would have enhanced the cost effective management of disease especially with limited resources in our society. A number of techniques have been purposed to be used as screening tools for OSAS, such as questionnaires and clinical models, utilizing symptoms, physical examination findings suggestive of OSAS, or different OSAS risk factors [9,10].

There are several OSAS questionnaires available as screening tools to identify patients at risk for OSAS. However, it is important to remember that these questionnaires are screening tools only and cannot replace a thorough history and physical or objective sleep-laboratory evaluation. The Berlin questionnaire (BQ) is one of the commonly known questionnaires for OSAS and has been validated in the primary care setting [11]. It has 10 questions organised into three categories. It incorporates questions about risk factors for sleep apnoea, such as snoring behaviour, wake-time sleepiness or fatigue, and the presence of obesity or hypertension. The BQ was originally developed in English and needs to be translated in other languages so that it can be applied in other countries and contexts. The aim of our study was to develop a Greek version of the BQ and to explore whether this screening questionnaire could be used to help identify primary care patients at greater risk of having OSAS, by comparing the BQ score with the apnoea–hypopnoea index (AHI) obtained from a formal sleep study.

## Methods

### Translation of BQ into Greek

After consent had been obtained from the developer of the BQ, it underwent a process of translation, back translation, and final review of obtained versions, according to the standard back translation methodology [12]. The questionnaire was translated into Greek by two bilingual translators working independently, thus generating two Greek versions, which were then translated back into English by another two bilingual individuals. The back translations were compared with the original English so that any necessary adjustments could be made, resulting in a single Greek BQ version that would be equivalent in meaning to the original (Additional file 1).

A cognitive debriefing was conducted by 2 independent interviewers with a group of 10 individuals, in order to test alternative wordings and to check the comprehensibility, interpretation, and cultural relevance of the translation.

### Patients

We performed a prospective study involving consecutive primary care patients, between 18 and 65 years old, who visited the General Hospital/Health Centre of Neapolis, Crete, Greece. Patients with no known history of any sleep disorder, competent to sign informed consent and agreeable to participating in the study were included. Patients with a prior diagnosis or treatment of OSAS, myocardial infarction, heart failure, stroke, neurological or psychiatric disorders, history of alcoholism, or upper respiratory tract infection within the past one week, were excluded, as were pregnant or critically ill patients, and subjects who did not consent to complete a sleep study, full clinical and anthropometric examinations, and the Greek version of the BQ. The group of patients that met inclusion criteria was then referred to the Sleep Disorders Unit, Department of Thoracic Medicine, University of Crete Medical School, between January and December 2011, for evaluation of suspected sleep-disordered breathing. All subjects provided written informed consent and ethical approval was obtained from both the University Hospital Ethics Committee and the General Hospital/Health Centre of Neapolis Scientific Board.

Study subjects underwent a detailed evaluation that included clinical history focused on sleep-related symptoms, associated conditions and comorbidities; they also completed the translated Greek BQ. Excessive daytime sleepiness was determined using the Epworth sleepiness scale (ESS) [13]. The results of the questionnaire were compared with polysomnography, which is considered the gold standard for OSAS diagnosis.

### Polysomnography

All patients underwent a single-night full diagnostic PSG according to standard techniques, with monitoring of the electroencephalogram (EEG) using frontal, central and occipital leads, electro-oculogram (EOG), electromyogram (EMG), flow (by oronasal thermistor and nasal air pressure transducer), thoracic and abdominal respiratory effort (induction plethysmography), oximetry, and body position. Snoring was recorded by a microphone placed on the anterior neck. Polysomnographic recordings were manually interpreted over 30-second periods, in accordance with the the new American Academy of Sleep Medicine (AASM) guidelines [14]. The scorer was always the same, blinded to the clinical condition of the patients and the previous results of the questionnaire. The determination of sleep stages and arousals was performed according to the AASM 2007 criteria, using EEG montages including frontal, central and occipital leads. The definition of apneas and hypopneas followed the AASM recommended criteria [14]. The AHI, calculated as the number of apnoea and hypopnoea events per hour of sleep was used for OSAS diagnosis and severity. OSAS was considered mild if the AHI was ≥5 per hour but <15 per hour, as moderate if AHI was ≥15 per hour but >30 per hour and as severe if AHI was >30 per hour.

### BQ score

The BQ contains 10 questions covering three categories including: 1) snoring severity (items 1–5), 2) excessive daytime sleepiness (items 6–9), and 3) history of high blood pressure or obesity (item 10). The questionnaire also includes information about age, sex, height, weight, and neck size. The patient is instructed to answer questions in all three categories, but the physician or medical staff is needed to analyse the responses. The BQ was scored (Additional file 1) as previously reported by Netzer and colleagues [11]. The first two categories are positive (one point is assigned) if responses indicate persistent symptoms (>3–4 times a week) on the questionnaire items. Item 5, about witnessed apnoeas, is an exception where a positive response is awarded two points. Category 2 includes an additional item concerning frequency of drowsiness behind the wheel (item 9) for which no point is assigned. Categories 1 and 2 are positive when the sum of all items is equal to or greater than 2, while category 3 is positive if the patient has high blood pressure and/or is obese (body mass index, BMI > 30). If the individual scores positive in at least 2 of the 3 categories, the patient is found to be at high risk for OSAS. However, if the patient scores positive in only one or none of the categories, then the patient is deemed to be at low risk for OSA.

### Statistical analysis

Basal characteristics were described as mean ± standard deviation for quantitative variables and as counts for proportions. Sensitivity, specificity, predictive values, likelihood ratio, odds ratio and their 95% confidence intervals, and the area under the curve (AUC) for each cut-off value of the diagnosis gold standard were calculated using standard methods by comparison with the PSG results. The Pearson correlation coefficient and level of significance were used to compare BQ score and risk groupings. The software SPSS 17.0.0 (Chicago IL, USA) was used. Probability values of p < 0.05 were considered as statistically significant.

## Results

### General characteristics of the population

We evaluated 189 primary care patients. Men made up 61.9% (n = 117) of the population, while 38.1% were women (n = 72). Ages ranged between 25 and 65 years (47 ± 13). The characteristics of the study population are given in Table 1. One hundred and three (54.5%) patients were obese (BMI > 30). Fifteen patients (7.9%) had a history of chronic obstructive pulmonary disease, 51 (26.9%) had systemic arterial hypertension and 16 (8.5%) diabetes mellitus.

**Table 1:** Characteristics of the study population (n = 189)

**Characteristics**	
Age, years	47 ± 13
Male gender, n (%)	117 (61.9)
Epworth sleepiness scale	10 ± 6
Hypertension (≥140/90 mmHg), n (%)	51 (26.9)
Body mass index (kg/m^2)^	35.0 ± 25.1
Normal (18.5 – 24.9), n (%)	30 (15.9)
Overweight (25 – 29.9), n (%)	56 (29.6)
Obese (≥30), n (%)	103 (54.5)

A PSG study was performed in 129 of the 189 subjects (68.3%). Further analysis was performed only in these 129 patients, and the remained 60 patients were excluded. The diagnosis of OSAS was confirmed in 91.5% of these 129 subjects (n = 118). Table 2 depicts PSG findings and Berlin Questionnaire findings in these 129 patients (Table 2).

**Table 2:** Polysomnography (PSG) findings and Berlin Questionnaire findings in the 129 patients who completed the PSG study

**PSG study**	
AHI, events per hour	41 ± 32
<5, n (%)	11 (8.5)
≥5– < 15, n (%)	20 (15.5)
≥15– ≤ 30, n (%)	30 (23.3)
>30, n (%)	68 (52.7)
Mean SaO_2_, %	92 ± 4
Minimum SaO_2_, %	80 ± 9
**Berlin Questionnaire (numbers of patients who were positive in each category)**	
Category 1, n (%)	117 (90.7)
Category 2, n (%)	54 (41.9)
Category 3, n (%)	97 (75.2)

SaO_2_: peripheral arterial oxygen saturation.

### Association between BQ score and AHI

BQ identified 74.4% (n = 96) of the patients as being at high risk of having OSAS and the remaining 25.6% (n = 33) as at low risk (Table 3). Table 3 shows the number of individuals with positive (high risk of sleep apnoea) and negative (low risk of sleep apnoea) scores on the BQ in each AHI range. For patients with a high risk score the OSAS diagnosis was confirmed by PSG study in 93.75% (n = 90). Thus, high-risk patients were more likely to have an ΑΗΙ of ≥ 5 and hence meet the criteria for OSAS. On the other hand, in those with a low-risk score, the OSAS diagnosis was excluded in only 15.2% (n = 5). Furthermore, a good positive and highly significant correlation was found between AHI and BQ score (Fisher’s exact test, p < 0.05), for AHI ≥ 15–≤ 30, meaning that the AHI scores were higher in the group identified as being at high risk of having sleep apnoea by the BQ.

**Table 3:** Relationship between Berlin questionnaire score and apnoea–hypopnoea index (AHI)

**AHI (n/h)**	**Low risk, n (%)**	**High risk, n (%)**
**<5**	5 (15.2)	6 (6.3)
**≥5– < 15**	14 (42.4)	6 (6.3)
**≥15– ≤ 30**	9 (27.3)	22 (22.9)
**>30**	5 (15.2)	62 (64.6)
**Total, n**	33	96

### BQ performance

Various cut-off values for AHI were used to stratify the patients in groups in relation to the severity of OSAS, as shown in Table 4. The sensitivity and the specificity of BQ for OSA diagnosis were 76% and 40%, respectively, for an AHI ≥ 5–< 15, 84% and 61% for an AHI ≥ 15–≤ 30, and 79% and 39% for an AHI > 30 (Table 4). The AUC obtained for AHI ≥ 15– ≤ 30 was 0.72 (95% CI: 0.64 – 0.80, p < 0.0001) on the Receiver Operating Characteristic (ROC) curve. This means that the probability that the BQ score predicted AHI ≥ 15–≤ 30 correctly was 72%. The AUC was only 0.58 (CI 95%: 0.49 – 0.67, p = 0.38) for detecting AHI ≥ 5–< 15 and 0.59 (CI 95%: 0.5 – 0.67, p = 0.13) for detecting AHI > 30.

**Table 4:** Predictive parameters for BQ

	**AHI ≥ 5 – <15 (95% CI)**	**AHI ≥ 15 – ≤ 30 (95% CI)**	**AHI > 30 (95% CI)**
**Sensitivity, %**	76 (67–73)	84 (76–91)	79 (69–86)
**Specificity, %**	40 (12–74)	61 (41–79)	39 (22–58)
**PPV, %**	94 (86–96)	86 (80–94)	80(71–87)
**NPV, %**	12 (3.5–28.2)	52 (34–69)	36 (20–55)
**Positive LR**	1.26 (0.6–27)	2.14 (1.6–2.9)	1.28 (0.8–2.0)
**Negative LR**	0.61 (0.3–1.1)	0.26 (0.1–0.5)	0.55 (0.3–0.9)
**Odds ratio**	2.069 (0.54–7.8)	8.21 (3.2–20.8)	2.31 (0.97–5.5)
**AUC**	0.578 (0.488–0.665)	0.724 (0.639–0.799)^*^	0.586 (0.496–0.672)

*AHI*: apnoea/hypopnoea index; *AUC*: area under the curve; *BQ*: Berlin Questionnaire; *LR*: likelihood ratio; *NPV*: negative predictive value; *PPV*: positive predictive value.

## Discussion

As the field of sleep medicine advances and more patients are diagnosed with OSAS and other sleep disorders, primary care clinicians will need to be able to recognise, diagnose, and treat these conditions. Recognizing sleep complaints is often challenging for both patients and clinicians. Although a polysomnogram is required to establish the diagnosis of OSAS, the long waiting lists for PSG in sleep centres have created interest in screening tools for obstructive sleep apnoea. While each of these has its own strengths and limitations, they all basically focus on similar high-risk features, such as habitual snoring, witnessed apnoeas, a high BMI, male sex, and advanced age. One validated tool that can help identify those with OSAS is the BQ. BQ is useful in screening sleep apnoea in a primary care population and may be more convenient and less costly than PSG.

Our investigation showed that, for different AHI cut-off points, BQ showed a moderate to high sensitivity (76–84%), a specificity that was low to moderate (40–61%), and a low positive likelihood ratio (1.26 to 2.14). However, positive predictive value is high (80–94%), indicating that if BQ is positive, for example at an AHI ≥ 5–< 15, there is a high (94%) likelihood that a person would actually have sleep apnea. We found a higher sensitivity and specificity when AHI was between 15 and 30. This finding suggests that in primary care patients, the Berlin questionnaire can be helpful in detecting a high risk of having OSAS, especially if the OSAS is moderate or severe.

The BQ is widely used as a screening tool for OSAS. It was an outcome of the Conference on Sleep in Primary Care in April 1996 in Berlin, Germany. The BQ is a standardised, self-administered questionnaire developed for assessing subjects at high risk for OSAS. It is inexpensive, easy to administer and has acceptable test–retest reliability [11,15,16]. This instrument has been used worldwide, such as in the USA [11], Jordan [17], and Nigeria [18]. In addition, it has been used for OSAS screening in surgical patients before anaesthesia [19], in patients undergoing endoscopy [20], as well as among orchestra members [21] or to predict outcomes after the catheter ablation of atrial fibrillation [22].

The predictive performance of the BQ varies among different patient populations. Our results are consistent with those reported by Netzer et al. [11], who also validated the BQ in a primary care population. The sensitivity and specificity obtained by Netzer and colleagues were 54% and 97%, respectively, at a cut-off of AHI ≥ 15 [11]. However, these sensitivity and specificity values have been criticised [15] as being erroneous, and it has been suggested that the actual sensitivity and specificity for Netzer’s study were 95% and 48%, respectively. Furthermore, in the Netzer manuscript, specificity increased as the AHI threshold increased and sensitivity fell. In our study, the sensitivity and specificity does not change very much at AHI ≥ 5, 15, 30, compared to the previous study. This may be due to several reasons. First, the population could be different. Polysomnography was offered to both high risk and lower risk patients, but more patients at high risk, who had sleep symptoms, selectively consented to the overnight polysomnography. Secondly, in the Netzer study, portable monitoring was used to assess the validity of the risk grouping strategy, compared to attended overnight polysomnography used in our study, which is the gold standard for the diagnosis of OSAS.

Apart from the Netzer study, previous studies exploring the BQ as an instrument to detect sleep apnoea showed similar or lower sensitivity and specificity compared to our results. The performance of the BQ in our population is in accordance with results obtained in other studies undertaken in a sleep laboratory in Portugal (72% and 50% for AHI ≥ 5– < 15, 82% and 45% for AHI ≥ 15– ≤ 30, and 88% and 39% for AHI > 30) [23], in a hypertension clinic (86% and 65% for AHI > 10) [24], and in preoperative patients (69% and 56% for AHI ≥ 5– < 15, 79% and 51% for AHI ≥ 15– ≤ 30, and 87% and 46% for AHI > 30) [19]. A better sensitivity of 86% and a specificity of 95% at a cut-off of AHI ≥ 5 were found when a modified version of the Berlin questionnaire was used [25] and in the Arabic version of the BQ (sensitivity of 97% and specificity of 90%) [26].

On the other hand, the results of our study differ from some others in the literature, where the BQ was shown to detect sleep apnoea in patients referred to a sleep laboratory with a lower sensitivity and specificity (68% and 49% for AHI ≥ 5, 62% and 43% for AHI ≥ 10, and 57% and 43% for AHI ≥ 15) [16], and in patients undergoing pulmonary rehabilitation (sensitivity and specificity 62.5% and 53.8% with a cut-off for AHI of 10 or greater) [27].

In our study, BQ demonstrated moderate to high sensitivity and low to moderate specificity: however, primary care practitioners require a practical and sensitive screening tool to identify patients at high risk of having OSAS. Because of the high prevalence of OSAS in primary care patients and the growing awareness of OSAS, general practitioners (GPs) are dealing with increasing numbers of patients with OSAS. Many patients who visit primary care physicians report risk factors, such as obesity and hypertension, and symptoms, such as snoring, sleepiness, and tiredness, suggestive of sleep apnoea. However, primary care providers often do not investigate these symptoms, and sleep apnoea frequently goes undiagnosed. Using the BQ as a screening tool for sleep apnoea in primary care population seems acceptable, being more convenient and less costly for health care users.

On the other hand, with the increased awareness of OSAS comes the risk that primary care physicians will refer patients with complaints of poor sleep, daytime sleepiness, and fatigue to sleep laboratories, without considering other diagnoses, such as insomnia, depression, and hypothyroidism [28,29]. Furthermore, excessive daytime sleepiness is a common complaint both in sleep-disordered patients and in a number of other non-medical and medical conditions, such as a shortage of sleep due to social or family reasons, shift working [30] or daytime non-shift working [31], and neurological disorders [32,33]. Therefore, given the need for stratification to determine patients who urgently require sleep studies, and in the face of rising health care costs and increased pressure for the more stringent control of health care resources, it would clearly be beneficial if primary care physicians could rely upon screening questionnaires to identify patients who are likely to have sleep apnoea, recommending sleep studies only for those patients who are at high risk of having a sleep disorder. In addition, most sleep clinics have long waiting lists, whereas patients who may have severe sleep breathing disorders warrant quicker diagnosis and treatment.

Our study presents some limitations that deserve comment. Firstly, there is a possible bias associated with the self-selection of patients. We found a high prevalence of OSAS in our group (91.5%), which is higher than estimates from community based surveys and is similar to the estimates found in surveys in sleep laboratories. As already mentioned, there may have been self-selection by the patients because those who had sleep symptoms might have selectively consented to the overnight polysomnography. Secondly, the mean BMI of the population in this study was high, with many patients being overweight and obese (29.6% and 54.5% respectively). The higher than average BMI of the sleep clinic population was likely to have affected the sensitivity and specificity of the BQ, as the BMI also factors heavily into the scoring of the BQ.

## Conclusions

In conclusion, our study confirms the importance of questionnaires in OSAS screening. These tools are inexpensive and easy to apply and should be used as a screening test in clinical practice. Due to the relatively high prevalence of undiagnosed OSAS and its complications for health, primary care clinicians need a reliable screening tool for OSAS prediction. Using the BQ, they could detect the possibility of OSAS during usual day practice visits and then identify high-risk groups of patients who should therefore be referred for further examination.

## Abbreviations

AASM: American Academy of Sleep Medicine; AHI: Apnoea–hypopnoea index; AUC: Area under the curve; BQ: Berlin Questionnaire; BMI: Body mass index; EEG: Electroencephalogram; EMG: Electromyogram; EOG: Electro-oculogram; ESS: Epworth sleepiness scale; GPs: General practitioners; LR: Likelihood ratio; NPV: Negative predictive value; OSAS: Obstructive sleep apnoea syndrome; PSG: Polysomnography; PPV: Positive predictive value; ROC: Receiver Operating Characteristic; SaO_2_: Peripheral arterial oxygen saturation.

## Competing interests

The authors declare that they have no competing interests.

## Authors’ contributions

IB, IDK and KM conceived the study, conducted the data collection, and participated in its design and in the data analysis. IB also wrote the manuscript. CM, MK, VM, EM, NMS and SES supervised the first author’s work and participated in its design and data analysis. All authors read and approved the final manuscript.

## Pre-publication history

The pre-publication history for this paper can be accessed here:

http://www.biomedcentral.com/1471-2466/13/6/prepub

## Supplementary Material

Additional file 1 Berlin questionaire. Click here for file
