# Supplementary material for: Translation and validation of Berlin questionnaire in primary health care in Greece
Source: BMC Pulm Med. 2013 Jan 24;13:6. doi: 10.1186/1471-2466-13-6 (PMC3561101; doi:10.1186/1471-2466-13-6)
Supplement: Additional file 1 — Berlin questionaire. [file 1471-2466-13-6-S1.pdf]

Ερωτηματολόγιο του Βερολίνου (Berlin Questionnaire)

Ύψος (m)_____ Βάρος (kg)_____ Ηλικία ______ Άρρεν / Θήλυ

Παρακαλούμε επιλέξτε την σωστή απάντηση σε κάθε ερώτηση.

ΚΑΤΗΓΟΡΙΑ 1

1. Ροχαλίζετε;
   ☐ α. Ναι
   ☐ β. Όχι
   ☐ γ. Δεν ξέρω

Αν ροχαλίζετε:

2. Το ροχαλητό σας είναι:
   ☐ α. Λίγο ηχηρότερο από την αναπνοή
   ☐ β. Όσο ηχηρό όσο η ομιλία
   ☐ γ. Δυνατότερο από την ομιλία
   ☐ δ. Πολύ δυνατό

3. Πόσο συχνά ροχαλίζετε;
   ☐ α. Σχεδόν κάθε μέρα
   ☐ β. 3-4 φορές την εβδομάδα
   ☐ γ. 1-2 φορές την εβδομάδα
   ☐ δ. 1-2 φορές το μήνα
   ☐ ε. Ποτέ ή σχεδόν ποτέ

4. Έχει ποτέ ενοχλήσει άλλους το ροχαλητό σας;
   ☐ α. Ναι
   ☐ β. Όχι
   ☐ γ. Δεν ξέρω

5. Παρατήρησε ποτέ κανείς ότι σταματάτε να αναπνέετε κατά τη διάρκεια του ύπνου;
   ☐ α. Σχεδόν κάθε μέρα
   ☐ β. 3-4 φορές την εβδομάδα
   ☐ γ. 1-2 φορές την εβδομάδα
   ☐ δ. 1-2 φορές το μήνα
   ☐ ε. Ποτέ ή σχεδόν ποτέ

ΚΑΤΗΓΟΡΙΑ 2

6. Πόσο συχνά νιώθετε κουρασμένος ή καταπονημένος μετά τον ύπνο;
   ☐ α. Σχεδόν κάθε μέρα
   ☐ β. 3-4 φορές την εβδομάδα
   ☐ γ. 1-2 φορές την εβδομάδα
   ☐ δ. 1-2 φορές το μήνα
   ☐ ε. Ποτέ ή σχεδόν ποτέ

7. Πόσο συχνά αισθάνεστε κουρασμένος, εξάντληση, ή ότι δεν αποδίδετε φυσιολογικά κατά τη διάρκεια της ημέρας;
   ☐ α. Σχεδόν κάθε μέρα
   ☐ β. 3-4 φορές την εβδομάδα
   ☐ γ. 1-2 φορές την εβδομάδα
   ☐ δ. 1-2 φορές το μήνα
   ☐ ε. Ποτέ ή σχεδόν ποτέ

8. Μισοκοιμηθήκατε ή αποκοιμηθήκατε ποτέ ενώ οδηγούσατε;
   ☐ α. Ναι
   ☐ β. Όχι

Εάν ναι:

9. Πόσο συχνά σας συμβαίνει αυτό;
   ☐ α. Σχεδόν κάθε μέρα
   ☐ β. 3-4 φορές την εβδομάδα
   ☐ γ. 1-2 φορές την εβδομάδα
   ☐ δ. 1-2 φορές το μήνα
   ☐ ε. Ποτέ ή σχεδόν ποτέ

ΚΑΤΗΓΟΡΙΑ 3

10. Έχετε υπέρταση;
   ☐ α. Ναι
   ☐ β. Όχι
   ☐ γ. Δεν ξέρω

Βαθμολόγηση βάσει του Ερωτηματολογίου του Βερολίνου

Προσαρμόστηκε από: Πίνακας 2 στο Netzer, et al., 1999 (Netzer NC, Stoohs RA, Netzer CM, Clark K, Strohl KP. Using the Berlin Questionnaire to identify patients at risk for the sleep apnea syndrome. Ann Intern Med. 1999 Oct 5;131(7):485-91).

Το ερωτηματολόγιο αποτελείται από 3 κατηγορίες που σχετίζονται με τον κίνδυνο εμφάνισης υπνικής άπνοιας. Οι ασθενείς δύναται να κατηγοριοποιηθούν σε ομάδες Υψηλού Κινδύνου και Χαμηλού Κινδύνου ανάλογα με τις απαντήσεις τους στις επιμέρους ερωτήσεις και την συνολική τους βαθμολόγηση στις κατηγορίες συμπτωμάτων.

Κατηγορίες και βαθμολόγηση:

Κατηγορία 1: ερωτήσεις 1, 2, 3, 4, 5.

Ερώτηση 1: εάν **«Ναι»**, προσδίδεται **1 βαθμός**
Ερώτηση 2: εάν η απάντηση είναι **«γ»** ή **«δ»** προσδίδεται **1 βαθμός**
Ερώτηση 3: εάν η απάντηση είναι **«α»** ή **«β»** προσδίδεται **1 βαθμός**
Ερώτηση 4: εάν η απάντηση είναι **«α»** προσδίδεται **1 βαθμός**
Ερώτηση 5: εάν η απάντηση είναι **«α»** ή **«β»** προσδίδονται **2 βαθμοί**

Προσθέστε τους βαθμούς. Η Κατηγορία 1 είναι θετική αν η συνολική βαθμολογία είναι ίση ή μεγαλύτερη του 2.

Κατηγορία 2: ερωτήσεις 6, 7, 8 (η ερώτηση 9 πρέπει να λαμβάνεται υπόψη ξεχωριστά).
Ερώτηση 6: εάν η απάντηση είναι **«α»** ή **«β»** προσδίδεται **1 βαθμός**
Ερώτηση 7: εάν η απάντηση είναι **«α»** ή **«β»** προσδίδεται **1 βαθμός**
Ερώτηση 8: εάν η απάντηση είναι **«α»** προσδίδεται **1 βαθμός**

Προσθέστε τους βαθμούς. Η Κατηγορία 2 είναι θετική αν η συνολική βαθμολογία είναι ίση ή μεγαλύτερη του 2.

Η Κατηγορία 3 είναι θετική αν η απάντηση στην ερώτηση 10 είναι «Ναι» Ή αν ο ΔΜΣ (Δείκτης μάζας σώματος) είναι μεγαλύτερος του 30kg/m^2.

(Πρέπει να υπολογιστεί ο ΔΜΣ. Ο ΔΜΣ ισούται με το πηλίκο του βάρους (kg) διά το τετράγωνο του ύψους (m), δηλαδή kg/m^2).

**Ομάδα Υψηλού Κίνδυνου:**	ανήκουν οι ασθενείς που βαθμολογούνται θετικά σε 2 ή περισσότερες κατηγορίες
**Ομάδα Χαμηλού Κινδύνου:**	ανήκουν οι ασθενείς που βαθμολογούνται θετικά σε 1 ή καμία κατηγορία

Πρόσθετη ερώτηση: πρέπει να λαμβάνεται υπόψη ξεχωριστά
